# (Mis)perception and Use of Unsterile Water in Home Medical Devices, PN View 360+ Survey, United States, August 2021^1^

**DOI:** 10.3201/eid2902.221205

**Published:** 2023-02

**Authors:** Shanna Miko, Sarah A. Collier, Claire E. Burns-Lynch, Ashley A. Andújar, Katharine M. Benedict, Julia C. Haston, Catherine O. Hough, Jennifer R. Cope

**Affiliations:** Centers for Disease Control and Prevention, Atlanta, Georgia, USA (S. Miko, S.A. Collier, C.E. Burns-Lynch, A.A. Andújar, K.M. Benedict, J.C. Haston, C.O. Hough, J.R. Cope);; Oak Ridge Institute for Science and Education, Oak Ridge, Tennessee, USA (C.E. Burns-Lynch);; Chenega Enterprise System & Solutions, Chesapeake, Virginia, USA (C.O. Hough)

**Keywords:** (Mis)perception, aerosolized tap water, contact lenses, tap water exposure, bacteria, nasal rinsing, unsterile water, sterile water, waterborne disease, enteric infections, home medical devices, biofilm, PN View 360+ Survey, United States

## Abstract

Tap water is not sterile, and its use in home medical devices can result in infections from waterborne pathogens. However, many participants in a recent survey in the United States said tap water could safely be used for home medical devices. These results can inform communication materials to reduce the high consequence of infections.

Tap water in the United States is treated to meet safe drinking standards; however, low levels of microorganisms remain in drinking water distribution systems, wells, and premise plumbing (*1**,**2*). Although most of these microorganisms are harmless and the water is safe for drinking and cooking, it might not always be safe for other uses, such as aerosolized inhalation and ocular or nasal irrigation (*2**–**4*). Microorganisms that can be found in water systems include *Pseudomonas aeruginosa*, nontuberculous mycobacteria (NTM), *Legionella* spp., *Acanthamoeba* spp., and *Naegleria fowleri* (*1**,**2*). In the United States, biofilm-associated pathogens such as *Pseudomonas* spp., NTM, and *Legionella* spp. are responsible for a large portion of the 120,000 hospitalizations, 7,000 deaths, and billions in direct healthcare costs annually related to waterborne diseases (*5*).

Persons who are at a higher risk for acquiring high-consequence opportunistic infections include the elderly, infants and young children, and persons who have weakened immune systems and other concurrent conditions (*2*,*6*). Some persons who have weakened immune systems might want to take precautions and use water free from microbes. Sterile water does not contain organic microbes but might contain inorganic materials, such as minerals; distilled water does not contain organic and inorganic materials (*7*).

At home, water is used for various health activities, including filling nasal rinsing devices and respiratory devices such as continuous positive airway pressure (CPAP) machines, vaporizers, and portable humidifiers. We hypothesize that people might not understand that tap water is not sterile, leading to its use for specific purposes, such as nasal rinsing, inhalation, and contact lens rinsing that are not recommended.

One study reported demographic data on household water use for home medical devices (*8*). Few data sources describe the understanding of sterile water sources by the population of the United States. This study aimed to quantify perceptions of sterile water, water sources, and actual use of water for home medical purposes among US adults and identify differences among demographic groups regarding perceptions and use of water. These findings can help focus educational efforts to increase awareness of safe water use practices for home medical activities, supporting healthcare providers and public health practitioners in advising their patients and communities about safe water practices for home medical activities.

## The Study

For this cross-sectional study, we used data from Porter Novelli Public Services and the ENGINE Insights’ PN View 360+ survey (https://styles.porternovelli.com), delivered August 16–18, 2021 (Appendix). Porter Novelli used quota sampling and statistical weighting to make the panel representative of the US population by sex, age, region, race/ethnicity, and education. All analyses were performed using SAS software version 9.4 (SAS Institute Inc., https://www.sas.com), and p<0.05 indicated significance. Survey procedures were used to assess the proportions of response variables by demographics. χ^2^ tests were conducted to test the association of each response variable with each demographic variable. Post hoc Wald F tests from contrast statements and the SurveyReg procedure SAS were used to compare subgroups for demographic variables with >2 levels.

The survey defined tap water as water from faucets and asked participants if they agreed or disagreed with a series of statements (Table 1). One-third (33%, 95% CI 30%‒36%) of respondents incorrectly answered that tap water does not have bacteria or living things present. Men, African American or Black persons, and urban residents were more likely to answer incorrectly. More than half (62%, 95% CI 59%‒66%) of participants said that tap water could be used for rinsing sinuses, 50% (95% CI 47%‒54%) for rinsing contact lenses, and 42% (95% CI 38%‒45%) for respiratory devices. Men and urban residents were more likely to choose >1 of these incorrect answers.

**Table 1:** Demographic characteristics of 1,004 respondents to question about potential uses for tap water, by response, in survey of knowledge about uses for tap water, PN 360 View 2021, United States*

Characteristic	Do you agree or disagree with the following statements about US tap water?				
	Can be used for drinking	Can be used for rinsing contact lenses	Can be used for nasal rinsing	Can be used in respiratory devices†	Bacteria and other living things are not present in US tap water
I don’t know	38	140	157	194	119
General population	81 (78‒83)	50 (47‒54)	62 (59‒66)	42 (38‒45)	33 (30‒36)
Sex					
F	77 (73‒81)	43 (38‒48)	58 (52‒63)	37 (32‒42	24 (20‒28)
M	84 (81‒88)	57 (52‒62)	67 (62,72)	46 (41‒51)	42 (37‒47)
p value	**0.0090**	**0.0002**	**0.0073**	**0.0153**	**<0.0001**
Race					
Black or African American	76 (68‒84)	47 (37‒57)	58 (48,68)	48 (37‒58)	48 (38‒58)
Other‡	69 (59‒78)	46 (35‒57)	57 (46,68)	35 (24‒45)	26 (17‒35)
p value	**0.0013**	0.5781	0.2917	0.2145	**0.0039**
Ethnicity					
Hispanic§	67 (58‒76)	48 (38‒58)	60 (51,70)	38 (28‒48)	31 (22‒40)
Non-Hispanic	83 (81‒86)	50 (47‒54)	63 (59,66)	42 (38‒46)	33 (30‒37)
p value	**<0.0001**	0.7034	0.6528	0.4189	0.6011
Age, y					
18–34	73 (68‒79)	50 (44‒56)	65 (59,71)	45 (38‒52)	38 (31‒44)
35–54	80 (75‒85)	52 (46‒58)	58 (52,64)	37 (31‒43)	35 (29‒40)
>55	86 (82‒90)	49 (43‒55)	64 (58,70)	43 (36‒49)	28 (23‒33)
p value	**0.0008**	0.7743	0.2784	0.1910	**0.0378**
Region					
Northeast	80 (74‒87)	59 (51‒67)	66 (57,74)	49 (41‒58)	34 (26‒41)
Midwest	85 (80‒90)	46 (38‒54)	60 (53,68)	41 (33‒49)	33 (26‒41)
South	82 (78‒86)	48 (42‒54)	63 (58,69)	40 (34‒45	34 (29‒39)
West	75 (68‒81)	51 (44‒58)	61 (54,68)	39 (32‒47)	30 (24‒37)
p value	0.0663	0.1199	0.7756	0.2810	0.8283
Metro status					
Urban	81 (76‒86)	59 (52‒65)	66 (60,72)	49 (43‒56)	44 (38‒50)
Suburban	79 (75‒83)	46 (40‒51)	59 (54,64)	38 (32‒43)	28 (24‒33)
Rural	83 (78‒88)	47 (40‒55)	64 (57,72)	39 (31‒46)	28 (21‒34)
p value	0.5218	**0.0052**	0.1933	**0.0163**	**<0.001**
Household income					
<$59,999	79 (76‒83)	48 (43‒53)	62 (57,66)	39 (35‒44)	31 (26‒35)
>$60,000	82 (78‒86)	53 (47‒58)	63 (58,68)	44 (39‒50)	36 (31‒41)
p value	0.2638	0.2013	0.6294	0.1722	0.0978
Water source¶					
Private well	90 (85‒96)	63 (54‒72)	71 (63‒79)	55 (46‒65)	48 (39‒57)
Municipal	81 (78‒84)	48 (44‒52)	61 (57‒65)	39 (35‒43)	30 (26‒34)
p value	**0.0101**	**0.0046**	0.0540	**0.0009**	**0.0002**

*Values are percentages agreeing (95% CIs) unless otherwise indicated. Boldface indicates significance (p<0.05 by χ^2^ or by Wald F-tests where there are >3 categories). “I don’t know” was not included in analysis; “Agree” and “Strongly Agree” were analyzed as “Agree”; “Disagree” and “Strongly Disagree” were not included in analysis. PN, Porter Novelli Public Services and the ENGINE Insights’ PN View 360+ Survey. †Respiratory devices include vaporizers, humidifiers, and continuous positive airway pressure machines. ‡Other race grouped persons who identified as >1 race, Asian, Native American, Alaska Native, or other because of small sample size. §Hispanic, Spanish, or Latino. ¶n = 932.

Respondents were asked how they used their household tap water (Table 2). Most persons reported using tap water for drinking, cooking, bathing, and handwashing. Approximately one quarter (24%, 95% CI 21%‒27%) of persons reported filling humidifiers or CPAP machines with tap water, 13% (95% CI 11%‒15%) reported using tap water for nasal rinsing, and 9% (95% CI 7%‒11%) reported using tap water for rinsing contact lenses.

**Table 2:** Demographic characteristics of 1,004 respondents to question about how they use tap water, by response, in survey of knowledge about tap water, PN 360 View 2021, United States*

Characteristic	How do you use your household tap water?					
	Consumption†	Bathing/ showering	Washing hands	Rinsing contact lenses	Nasal rinsing	Filling respiratory devices‡
General population	66 (63‒69)	84 (82‒86.4)	88 (86‒90)	9 (7‒11)	13 (11‒15)	24 (21‒27)
Sex						
F	64 (59‒68)	83 (80‒87)	87 (84‒90)	8 (5‒10)	12 (9‒15)	24 (21‒28)
M	68 (63‒72)	85 (81‒88)	89 (86‒92)	10 (7‒13)	14 (11‒17)	23 (20‒27)
p value	0.2140	0.6004	0.3666	0.3034	0.3902	0.7322
Race						
White	71 (68‒74)	87 (84‒89)	89 (87‒92)	9 (7‒11)	13 (11‒16)	24 (21‒27)
Black or African American	53 (44‒62)	77 (70‒85)	85 (78‒91)	10 (5‒16)	10 (5‒,15)	22 (15‒30)
Other§	52 (42‒62)	77 (69‒86)	83 (75‒91)	8 (3‒14)	14 (7‒20)	24 (15‒32)
p value	**<0.0001**¶	**0.0078**¶	0.0814	0.8320	0.5920	0.9130
Ethnicity						
Hispanic#	51 (42‒61)	71 (63‒79)	78 (70‒86)	13 (7‒19)	21 (14‒29)	25 (17‒33)
Non-Hispanic	69 (65‒72)	87 (84‒89)	90 (88‒92)	8 (6‒10)	11 (9‒13)	24 (21‒27)
p value	**0.0003**	**<0.0001**	**0.0003**	0.0813	**0.0012**	0.7937
Age, y						
18–34	57 (51‒63)	73 (67‒78)	81 (76‒86)	14 (10‒18)	19 (14‒24)	25 (20‒30)
35–54	64 (58‒69)	83 (79‒88)	87 (83‒91)	10 (7‒14)	13 (10‒17)	25 (20‒30)
>55	74 (70‒79)	93 (90‒96)	94 (9‒97)	4 (2‒5)	8 (5‒11)	22 (18‒26)
p value	<0.0001**	**<0.0001**††	<0.0001**	<0.0001**	0.0002**	0.5591
Region						
Northeast	70 (63‒77)	82 (76‒88)	89 (84‒94)	10 (5‒14)	12 (7‒16)	31 (24‒38)
Midwest	69 (63‒76)	84 (78‒89)	85 (80‒91)	8 (4‒12)	12 (8‒17)	27 (21‒34)
South	66 (61‒71)	87 (84‒91)	89 (86‒93)	8 (5‒11)	12 (8‒15)	18 (14‒22)
West	58 (52‒65)	81 (75‒86)	87 (82‒91)	10 (6‒15)	15 (11‒20)	25 (19‒31)
p value	0.0601	0.2120	0.5344	0.7265	0.5647	**0.0054**‡‡
Community setting						
Urban	62 (56‒67)	82 (78‒87)	88 (84‒2)	13 (9‒17)	16 (12‒20)	24 (19‒28)
Suburban	65 (60‒69)	83 (79‒87)	86 (83‒90)	8 (5‒10)	13 (10‒16)	24 (20‒28)
Rural	74 (68‒80)	89 (85‒93)	91 (86‒95)	6 (3‒9)	7 (4‒11)	24 (18‒30)
p value	**0.0189**§§	0.1004	0.3463	**0.0115**¶¶	**0.0147**§§	0.9797
Household income						
<$59,999	63 (59‒67)	83 (80‒87)	86 (83‒89)	7 (5‒10)	9 (7‒12)	20 (17‒24)
>$60,000	69 (65‒74)	85 (82‒89)	90 (87‒93)	11 (8‒14)	17 (14‒21)	29 (25‒34)
p value	0.0608	0.4593	0.0837	**0.0355**	**0.0003**	**0.0024**
Water source, n = 932						
Private well	68 (61‒76)	76 (69‒83)	82 (75‒88)	14 (8‒19)	18 (12‒24)	38 (29‒46)
Municipal	67 (63‒70)	87 (84‒89)	90 (87‒92)	8 (6‒10)	12 (10‒14)	22 (19‒25)
p value	0.6712	**0.0016**	**0.0102**	**0.0179**	**0.0388**	**0.0001**

*Values are percentages (95% CIs) unless otherwise indicated. Boldface indicates significance (p<0.05 by χ^2^ or by Wald F-tests where there are >3 categories; specific comparisons are footnoted). PN360 View, Porter Novelli Public Services and the ENGINE Insights’ PN View 360+ Survey. †Includes drinking, rinsing produce, or making ice. ‡Respiratory devices include vaporizers, humidifiers, and continuous positive airway pressure machines. §Other race grouped persons who identified as >1 race, Asian, Native American, or Alaska Native, or other because of small sample size. ¶For comparison between White and Black or African American and between White and other race categories. #Hispanic, Spanish, or Latino. **For comparisons between 18–34-y and >55-y age groups and between 35–54-y and >55-y age groups. ††For comparisons between 18–34-y and 35–54-y age groups, between 18–34-y and >55-y age groups, and between 35–54-y and >55-y age groups. ‡‡For comparisons between Northeast and South, between Midwest and South, and between South and West. §§For comparisons between urban and rural and between suburban and rural. ¶¶For comparisons between urban and suburban and between urban and rural.

## Conclusions

The results of this survey highlight opportunities to reinforce messaging regarding appropriate uses of tap water and recommendations for using water in medical devices at home. Although most persons understand what sterile water is and acknowledge that tap water is not sterile, a large proportion of persons responded that tap water can be used for nasal rinsing devices, contact lens rinsing, and filling respiratory devices. These findings represent an opportunity for public health practitioners and water utilities to continue communicating the value of US tap water and its appropriate use.

Although waterborne opportunistic infections can occur through multiple routes and can depend on a person’s health status, most NTM and *Legionella* infections are acquired through inhalation (*9*). In this study, 24% of respondents reported filling respiratory devices with tap water, consistent with a recent study in which 20% of respondents reported filling respiratory devices with tap water (*8*). Studies have demonstrated measurable concentrations of NTM (*9*), concentrated minerals (*10*), and other contaminants in aerosols from humidifiers (*11*). Water quality affects the quality of aerosolized air emitted from humidifiers, CPAP machines, and vaporizers, underlying the need for sterilized or distilled water to be used in those devices. Healthcare providers and pharmacists are uniquely positioned to share additional recommendations on appropriate waters (i.e., sterile, distilled, or boiled and cooled) for respiratory devices when they are sold or prescribed. Persons should be informed that they can reduce their exposure to waterborne pathogens by using distilled water or water that has been appropriately boiled and cooled and by regularly cleaning and disinfecting all respiratory devices that use water.

Water can be sterilized at home for safe home medical use for respiratory devices and neti pots (containers designed to rinse debris or mucus from the nasal cavity). Ordemann et al. tested water treatment options of UV light treatment, granular activated carbon filtration, and boiling to eliminate *Staphylococcus aureus*, *Pseudomonas aeruginosa*, *Moraxella catarrhalis*, *Acinetobacter baumannii*, *Klebsiella pneumoniae*, *Legionella pneumophila*, and *N. fowleri.* They reported that sterilization could be achieved by boiling water for 5 minutes then cooling, or by UV treatment (e.g., SteriPEN, https://www.katadyngroup.com) for 45 seconds or following the manufacturer’s instructions (*12*). The Centers for Disease Control and Prevention also has recommendations for preparing safer water for nasal rinsing, which includes boiling for 1 minute (3 minutes at elevations >6,500 feet) and cooling (*13*). Focused public health messaging and communications from health departments, pharmacists, and healthcare providers should increase awareness of how best to achieve sterile water at home for those who need it, reducing the number of biofilm-associated waterborne pathogens persons are exposed to when performing home medical activities.

Biofilm-associated waterborne pathogens make up a substantial portion of waterborne disease-related illnesses and deaths in the United States. Our results indicate demographic groups to which future public health and provider efforts should be directed to promote appropriate household tap water management practices when using home medical devices that aerosolize water or irrigate the eyes and nose. Public health messaging and healthcare provider guidance that incorporates risk factors for these device users and aligns with recommendations of the Centers for Disease Control and Prevention (*2*) are effective risk communication strategies that can influence population behavior change.

Appendix Additional information on (mis)perception and use of unsterile water in home medical devices, PN View 360+ Survey, United States, August 2021.
