# Supplementary material for: (Mis)perception and Use of Unsterile Water in Home Medical Devices, PN View 360+ Survey, United States, August 2021
Source: Emerg Infect Dis. 2023 Feb;29(2):397–401. doi: 10.3201/eid2902.221205 (PMC9881759; doi:10.3201/eid2902.221205)
Supplement: Appendix — Additional information on (mis)perception and use of unsterile water in home medical devices, PN View 360+ Survey, United States, August 2021. [file 22-1205-Techapp-s1.pdf]

Article DOI: DOI: https://doi.org/10.3201/eid2902.221205

(Mis)perception and Use of Unsterile Water in Home Medical Devices, PN View 360+ Survey, United States, August 2021

Appendix

Methods

PN View is a rapid turnaround opt-in internet panel survey that provides insights into the knowledge and practices of specific audiences. Panel members who had not completed a survey in the previous 20 waves of survey administration were eligible to participate. Participants were asked questions regarding their source of daily household water, perceptions of US tap water, daily water use, and understanding of sterile water. Of the 2,373 respondents ≥18 years of age who started the survey, 1,004 (42.3%) were not over their quota and could complete the survey. Participants were provided an incentive to encourage participation. They were informed that their answers were being used for market research and that they could refuse to answer any question at any time. No personally identifying information was included in the data files. Weights were designed to match Current Population Survey proportions from the US Census Bureau. The PN View 360+ survey included nine demographic variables: gender, age, race, ethnicity, education, household income, household size, metropolitan status, and census region. The following demographic variables and mutually exclusive categories were created for our weighted analysis: gender (male/female); age (18–34, 35–54, and 55 years of age and older); race (White, Black, “other” race). CDC licensed the data from Porter Novelli Public Services. Although Porter Novelli Public Services and its vendors are not subject to CDC IRB review, they adhere to all professional standards and codes of conduct set forth by the Council of American Survey Research Organizations.

Results

Perceptions of Sterile Water

When asked to choose the one response that best described their understanding of the term “sterile water,” 63% (95% CI 60,66) of respondents correctly answered that ‘sterile water’ refers to water that does not contain any bacteria or other living organisms.” More respondents aged 55 years or older (70%; p = 0.0010) answered correctly compared with other age groups.

Few respondents (12% (10,14)) believed that the water that comes out of their faucets was sterile (Appendix Table 2). Persons living in urban areas and persons younger than 55 years were more likely to believe this. A small portion of persons answered that sterile water is not needed for contact lens rinsing (12% (10,14)) or sinus rinsing (15% (13,17)); in both responses, a greater proportion of men selected these incorrect options.

Uses of Tap Water

Table 2 describes more respondents who identified as Hispanic (e.g., Hispanic, Spanish, or Latino) descent reported using tap water for nasal rinsing (21%, p = 0.0012) than non-Hispanic respondents (11% (9,13)). Fewer participants aged 55 years and older reported using tap water to rinse contact lenses (4%, p<0.0001) and to rinse sinuses (8%, p = 0.0002) than those in other age groups. Fewer persons living in the South reported using tap water to fill respiratory devices (18%, p = 0.0054). A higher proportion of persons who had an annual income of more than $60,000 reported the use of tap water to fill respiratory devices (29%, p = 0.0024), rinse contact lenses (11%, p = 0.0355) and sinuses (17%, p = 0.0003) compared with those with an income below $60,000. More respondents in urban areas reported using tap water for rinsing contact lenses (13%, p = 0.0115) compared with those living in other community settings, and fewer persons in rural areas reported using tap water for nasal rinsing (7%, p = 0.0147) compared with those living in other community settings. In all the medical device use categories, more private well water users reported using their household tap water for contact lens rinsing (14%, p = 0.0179), nasal rinsing (18%, p = 0.0388), and respiratory devices (38%, p = 0.0001) than municipal water users.

Limitations

The findings of this analysis are subject to several limitations. First, survey responses were self-reported and asked at a single point in time. Due to recall error and reporting bias,

responses may not accurately reflect respondents' actual tap water use. Second, while this survey was weighted to be representative of the US population, small sample sizes within subgroups made it challenging to generate exact estimates for certain populations. Third, respondents who were asked whether they used tap water for respiratory and nasal rinsing devices could not specify if they sterilized the water before use. It is possible that some respondents properly sterilized the water before use. Conversely, those who reported using sterile water for home medical devices may not be doing so because analysis shows an apparent misunderstanding of what constitutes sterile water. In addition, questions asked were about water for *filling* these devices and not for cleaning. Although using sterile water for filling is recommended to reduce exposure to biofilm-associated organisms, using unsterilized water for cleaning or rinsing devices and not enabling the device to dry completely can promote the growth of pathogens. Furthermore, we could not assess how many respondents wore contact lenses, used CPAP machines or humidifiers, or rinsed their sinuses because this data was not collected during the initial survey, thus limiting the utility of the comparison group who answered negatively. Despite these limitations, this cross-sectional study of population perceptions and use of water at home fills a gap in the literature and demonstrates a misunderstanding of sources of sterile water resulting in the misuse of water for home medical devices.

Appendix Table 1. Unweighted demographic characteristics of all respondents – United States, PN 360 View 2021 (n = 1,004)

Characteristic	Unweighted frequency (%)
Sex	
Female	502 (50)
Male	502 (50)
Race	
White	786 (78)
Black or African American	119 (12)
Other*	99 (10)
Ethnicity	
Hispanic†	116 (12)
Non-Hispanic	888 (88)
Age, y	
18–34	298 (30)
35–54	348 (35)
55+	358 (36)
Region	
Northeast	187 (19)
Midwest	206 (21)
South	380 (38)
West	231 (23)
Community setting	
Urban	320 (32)
Suburban	459 (46)
Rural	225 (22)
Household income	
Less than $59,999	567 (56)
$60,000 or more	437 (44)
Water source	

Characteristic	Unweighted frequency (%)
Private well	148 (15)
Municipal water	784 (78)
Cisterns	24 (2)
I don't know	48 (5)

*Other race grouped persons who identified as more than one race, Asian, or Native American or Alaska Native, or other due to small sample size.
†Hispanic, Spanish, or Latino.

Appendix Table 2. Demographic characteristics of respondents for "Select all responses you believe to be true regarding sterile water." – United States, PN 360 View 2021 (n = 1,004)

Characteristic	Water from the faucets in my home is sterile Weighted% (95% CI)	Does not need to be sterile for drinking	Does not need to be sterile to rinse contact lenses	Does not need to be sterile for nasal rinsing
General population	12 (10,14)	27 (24,30)	12 (10,14)	15 (13,17)
Sex				
a. Female	9 (7,12)	26 (22,30)	9 (6,11)	11 (8,14)
b. Male	14 (11,17)	28 (24,32)	16 (12,19)	19 (15,23)
p-value	0.0333	0.5969	0.0014	0.0005
Race				
c. White	11 (8,13)	30 (26,33)	12 (10,14)	14 (12,17)
d. Black or African American	13 (7,19)	17 (10,24)	10 (4,15)	15 (9,22)
e. Other*	15 (8,22)	23 (14,31)	14 (7,21)	18 (10,26)
p-value	0.3413	0.0167; c-d	0.6080	0.5743
Ethnicity				
f. Hispanic†	15 (9,22)	19 (11,26)	19 (11,26)	17 (10,24)
g. non-Hispanic	11 (9,13)	28 (25,32)	11 (8,13)	14 (12,17)
p-value	0.1312	0.0337	0.0123	0.39
Age				
h. 18–34	17 (12,21)	18 (13,22)	13 (9,18)	18 (13,22)
i. 35–54	13 (10,17)	26 (21,31)	12 (8,15)	14 (10,18)
j. 55+	6 (4,9)	35 (29,40)	11 (8,14)	13 (10,17)
p-value	0.0002; h-j, i-j	<0.0001; h-i, h-j	0.6834	0.3083
Region				
k. Northeast	9 (5,13)	34 (26,41)	13 (7,18)	15 (10,21)
l. Midwest	11 (7,15)	25 (19,31)	13 (8,18)	13 (8,18)
m. South	11 (8,15)	27 (22,31)	12 (8,15)	15 (11,19)
n. West	14 (10,19)	24 (18,30)	11 (7,15)	15 (11,20)
p-value	0.4018	0.1887	0.9480	0.8797
Community setting				
o. Urban	17 (13,21)	19 (15,24)	14 (10,18)	16 (12,20)
p. Suburban	10 (7,13)	28 (24,32)	11 (8,15)	15 (11,18)
q. Rural	7 (4,10)	36 (29,42)	10 (6,15)	14 (9,18)
p-value	0.0014; o-p, o-q	0.0002; o-p, o-q	0.4797	0.8105
Household income				
r. Less than $59,999	10 (7,12.6)	23 (19,27)	11 (8,13)	12 (9,15)
s. $60,000 or more	14 (11,17.0)	32 (28,37)	14 (10,17)	18 (15,22)
p-value	0.0693	0.0014	0.1822	0.0101
Water source§				
t. Private well	17 (11,23)	28 (20,36)	13 (7,18)	18 (12,25)
u. Municipal water	10 (8,12)	28 (25,31)	12 (10,14)	15 (12,17)
p-value	0.0200	0.9721	0.8599	0.2846

Letters indicate significant chi-square p-values or Wald F-tests where there are three or more categories.
*Other race grouped persons who identified as more than one race, Asian, or Native American or Alaska Native, or other due to small sample size.
†Hispanic, Spanish, or Latino.
§n = 932.
